# Organoleptic Evaluation of Amomi Fructus and Its Further Background Verified via Morphological Measurement and GC Coupled with E-Nose

**DOI:** 10.1155/2018/4689767

**Published:** 2018-03-05

**Authors:** Dong Xu, Yan Lin, Rudolf Bauer, Hui-Rong Chen, Rui-Qi Yang, Hui-Qin Zou, Yong-Hong Yan

**Affiliations:** ^1^School of Chinese Materia Medica, Beijing University of Chinese Medicine, Beijing 100029, China; ^2^Institute of Pharmaceutical Science, University of Graz, 8010 Graz, Austria

## Abstract

The present study investigated the maneuverability and reasonability of sensory analysis, which has been applied in TCM identification for a long time. Ten assessors were trained and generated the human panel to carry out the organoleptic evaluation of twenty-five batches of Sha-Ren samples. Accordingly, samples were scored from 0 (lowest) to 10 (highest) for sensory attributes. Based on this, samples were divided into three classes: high class (Yang-Chun-Sha from Guang-Dong), moderate class (Yang-Chun-Sha samples from Yun-Nan and Guang-Xi), and low class (Lv-Qiao-Sha from marketplaces). For further background, three instrumental approaches were employed: morphological measurement with three indices (longitudinal diameter, transverse diameter, and 100-fruit weight), GC for determination of bornyl acetate contents, and E-nose for aromatic fingerprint. It is demonstrated in the results that GC and E-nose analyses were in great agreement with organoleptic evaluation. It gives insights into further studies on searching better morphological indicators and improving discriminant model of E-nose.

## 1. Introduction

Amomi Fructus, called “Sha-Ren” in Chinese, has been one of the commonly used herbs in Traditional Chinese Medicine (TCM) for more than 1,300 years, mainly for the treatment of gastrointestinal diseases. Besides, its widespread application in food industry also gains much attentions, not only in China, but in other Southeast Asian countries, including Thailand, Vietnam, Burma, Indonesia, and so forth. Its yearly consumption reaches more than 3.1 million kg and maintains a constant increasing trend, which reflects strong market demands and potential commercial opportunities. However, due to confusable species of Sha-Ren from different producing areas, its price varies remarkably more than a hundredfold. Therefore, it is urgent and necessary to develop a rapid and reliable approach to distinguish Sha-Ren from different classes. On the other hand, the adulteration also needs cautiousness, especially in clinical use, because incorrect or fake herbal medicines could result in low clinic effect or even poisoning. Many qualitative and quantitative methods have been employed in quality assessment of Sha-Ren from different species and habitats, for instance, GC-MS and IR analysis [1, 2]. However, those conventional physicochemical assays are mostly time-consuming, expensive, of high technical skills, and not so practical.

Macroscopic identification, as one of the five typical methods for TCM authentication [3–5], has been acknowledged to be a simple, convenient, and efficacious approach. During its procedure, morphological characteristics (shape, weight, length, etc.) and sensory properties (odor, taste, etc.) are carefully selected, observed, recorded, and analyzed, which help well-trained experts to do a quick and good job in marketing test of TCM [6–8]. Nevertheless it cannot not be neglected that the obvious drawbacks of this method are low repeatability and imprecision and depending on the experience of the estimators to a great extent. Therefore, this leaves us with a serious of problems: Which morphological characteristics should be chosen to be valuable indices? What is the slight discrepancy among samples applying smelling, tasting, and touching senses? How to define the comparability of results from different experts, whom to trust, and whom not to? All in all, though this macroscopic identification seems to be a promising method for TCM quality evaluation, we firstly have to seek out the solutions to all the problems mentioned above.

The unique advantage of organoleptic analysis benefits from its capability of offering information perceived via human senses stimulating by a complex set of chemical compounds. For instance, odor is mainly perceived by the interactions of the volatiles with the olfactory epithelium in our nasal cavity [9]. Therefore, it could be a feasible way to introduce instrumental analysis as an additional supplementation, which helps to overcome the disadvantages of organoleptic analysis and also gives further background to demonstrate the material basis. Proper instrumental techniques permit detailed and objective information of human senses. Instead of vague odor from smelling, GC and electronic nose (E-nose) could provide a detailed and holistic odor profile, respectively. The former gives information concerning individual compounds and the latter concerning the origin, nondestructive, and holistic aromatic fingerprint. These two techniques have been employed in many aromatic researches and both got reliable results, separately or jointly [10–12]. However, the number of reports focused on combining these two instrumental techniques with morphological measurement and illustrating the basis of organoleptic evaluation from the material aspect is still limited, especially involving TCM.

In Pharmacopoeia of People's Republic of China [13], the dried fruits of three species from* Zingiberaceae* family are recorded as legal origins of Sha-Ren: (I)* Amomum villosum* Lour., (II)* Amomum villosum* Lour. var.* xanthioides* T. L. Wu et Senjen, and (III)* Amomum longiligulare* T. L. Wu. However, according to our previous investigations, the first (Yang-Chun-Sha in Chinese) and second (Lv-Qiao-Sha in Chinese) have been mainstream products whilst the third (Hai-Nan-Sha in Chinese) has not been easily pursued and collected in trading markets. Besides, back to the original habitat of the third, namely, Hai-Nan province, its plantation has been in a poor state for many years. On the contrary, the first one is well-known with its high quality and considered as genuine TCM from famous regions in Guang-Dong province. Even more noteworthy is that in 2005 Yang-Chun-Sha was nominated as one of 1992 China protected geographical indication products [14], among them 146 products locate themselves in Guang-Dong province, which means Yang-Chun-Sha representing high quality. As a result, Yang-Chun-Sha's price occupies the highest. Many researches have been carried on to find its unique indices and protect its correct usage [15, 16]. The contents of volatile constituents of Yang-Chun-Sha play a significant role in the authentication as well as its particular odor.

In the view of pros and cons of each technique, there are no universal and perfect methods for the simultaneous analysis of every volatile compound and the particular odor, which is essential for the development of distinguishing Sha-Ren rapidly and reliably. To the best of our literature survey, no study on organoleptic analysis of Sha-Ren coupled with GC and E-nose has been reported to date. Therefore, this study herein aims (1) to establish a practical and precise organoleptic method for identification of Sha-Ren, (2) to replenish sensory odor with detailed information of volatile compounds and digitalized aromatic fingerprint from GC and E-nose, and (3) to offer high-priced Sha-Ren material background and shed light on its reasonability.

## 2. Materials and Methods

### 2.1. Chemicals and Reagents

Bornyl acetate standard (batch number 110759-200604) was purchased from National Institutes for Food and Drug Control (Beijing, China). Absolute ethyl alcohol and other reagents used were all at chromatographic grade and from Dikma Technologies Co., Ltd. (Beijing, China).

### 2.2. Sha-Ren Samples

A total of 25 batches of Sha-Ren were collected: 19 batches of them were Yang-Chun-Sha from three habitats (Guang-Dong, Yun-Nan and Guang-Xi) and the rest 6 batches of them were Lv-Qiao-Sha from marketplaces. As mentioned above, the third species Hai-Nan-Sha were not obtained. All the samples have been authenticated via Professor Yong-Hong Yan from Department of Chinese Materia Medica of Beijing University of Chinese Medicine. They were packed in sealable plastic bags separately and stored at 4°C until analysis.

### 2.3. Organoleptic Evaluation

Based on observing, touching, and sniffing senses, organoleptic analysis was carried out by a human panel with ten assessors [17, 18], consisting of 10 trained postgraduate students of Beijing University of Chinese Medicine. The samples were evaluated by the panelists one by one, who were also placed in separate labs for unbiased analysis. Sha-Ren samples were taken out and presented at room temperature for at least two hours. They were scored from 0 (lowest) to 10 (highest) for sensory attributes of appearance, texture, and odor.

### 2.4. Morphological Measurement

Three objective indices were chosen to represent the morphological characteristics: longitudinal diameter (LD), transverse diameter (TD), and 100-fruit weight (100 FW). According to published reports and experienced experts, these three indices summarized the size of Sha-Ren and expressed the extent of oiliness, which are closely related to the appearance and texture of Sha-Ren. What is more, these three could be measured into figures, which helped us conduct further analysis.

Method of coning and quartering were employed to take 20 Sha-Ren fruits. Then LD and TD values were measured by vernier caliper (Measuring Instrument & Cutting Tool Factory, Beijing, China) and afterwards the mean was calculated.

Also using method of coning and quartering but to take 100 Sha-Ren fruits, then 100 FW value was measured by BS-124S electronic analytical balance (Sartorius, Germany) in triplicate and the means was calculated as well.

All values should not be utilized until RSD < 3%.

### 2.5. GC Analysis

According to previous reports, bornyl acetate is one of the main active volatile compounds [19, 20] in Sha-Ren and also one of the legal examination indicators in Chinese pharmacopoeia. Therefore, in this paper, bornyl acetate was chosen to be the index of detailed aromatic information and help supplement the results of sensory analysis.

Bornyl acetate standard was weighed precisely and dissolved in absolute ethyl alcohol to 11.90 mg/mL.

Around 1 g of pulverized Sha-Ren samples was added to a conical flask with 25 mL absolute ethyl alcohol and extracted with ultrasonic at 40 kHz for 30 min. After cooling down to the room temperature and compensating weight, the processed samples were filtered and stored at 4°C until analysis.

The GC system included an Agilent 7890A instrument (California, America) with a flame ionization detector (FID): GC conditions: Agilent DB-1 column, (0.25 *μ*m, 0.25 mm × 30 m; Quartz Capillary); carrier gas: nitrogen; flow rate: 1.3 mL/min; the column oven temperature: 100°C;tThe injection temperature: 230°C; the FID temperature: 250°C; split ratio: 10 : 1; the injection volume: 1 *μ*L. The linearity of this method was assayed by analyzing standard solutions in the range of 0.1190–0.8333 mg/mL for bornyl acetate.

### 2.6. E-Nose Analysis

*α*-Fox 3000 E-nose (Alpha MOS, Co., Ltd., France) with 12 metal-oxide gas (MOG) sensors was employed to obtain the digitalized aromatic fingerprint for the assessment of the odor strength. This E-nose instrument contains three main parts: an automatic sampling system, namely, HS-100 sampler, two chambers with six MOG sensors in each, and a data acquisition unit connected to a computer, which allows data storage and processing. The materials and properties of these 12 MOG sensors have been reported in our previous researches as well as the optimization method [21]. The whole 12 MOG sensors consist of (i) six LY-type sensors including LY2/LG, LY2/G, LY2/GH, LY2/AA, LY2/gCT, and LY2/gCTL (majorly sensitive to short chain volatile fatty acids and aldehydes); (ii) four P-type sensors including P10/1, P10/2, P40/1, and PA/2 (majorly sensitive to methane, propane, and other aliphatic nonpolar molecules), and (iii) two T-type sensors including T30/1 and T70/2 (majorly sensitive to polar alcoholic and chlorinated compounds), which allows E-nose perceived responses of most volatile compounds [22]. All 12 MOG sensors were named from S1 to S12.

Sha-Ren samples were firstly ground and sifted through a mesh of 850 *μ*m; then 0.4 g of powder was sealed into a glass and underwent predefined thermoincubation, which was vital to facilitate the generation and kinetic equilibrium of headspace volatile compounds inside the glass. Secondly the upper air containing particular volatile compounds was injected into the *α*-Fox 3000 E-nose system via the HS-100 autosampler applying pure air as the carrier gas at a flow rate of 150 mL/min. The incubation conditions were programmed at 45°C for 600 seconds with 250 r/s rotating speed. The temperature and volume of injection were set to 50°C and 500 *μ*L.

The volatile compounds in the upper air from the sealed glass interacted with the MOG sensors and resulted in changes of their resistance, positively or negatively, which were recorded in the computer. The relative change in their resistance (Δ*R*/*R*_0_) value was monitored precisely per second for 200 s followed by the dormancy period of 600 s to ensure that the sensors must return to the base line and avoid their poisoning. The output Δ*R*/*R*_0_ values were plotted against time. In that case, 12 MOG sensors lasting 200 seconds brought every sample a huge data array of 2400 values. Herein in our research, the absolute value of maximum sensor response was selected and analyzed to generate the holistic aromatic fingerprint of every sample. The test of each sample was repeated six times.

### 2.7. Statistical Analysis

Friedman test, one of nonparametric tests, was introduced to analyze the 25 related samples and compare the consistency of the organoleptic evaluation by 20 panelists. Then logistics regression analysis with ordinal variables was employed to find out the differences of sensory characteristics among the samples from different species and habitats.

For data processing of morphological measurement, hierarchical cluster analysis (HCA) and discriminant analysis (DA) were both utilized to see how many groups those all 25 batches samples were divided into and whether it was in the agreement with organoleptic evaluation.

In the past decades, E-nose has been used in many fields and well-known as a promising approach for fast and noninvasive detection [23, 24]. However, lack of unified and efficacy data analysis for its discriminative model has become the bottleneck to a great extent. Theoretically, it could be more than just one pattern recognition based on the rapid development of mathematical statistics. However, searching the optimum strategy becomes an inevitable problem. Our team figured out two solutions to improve the discriminative model and increase the distinguishing positive rate, concerning BestFirst + CfsSubsetEval (BC) feature extraction, and cascade classifier [21, 25, 26]. Therefore, as proven to be an efficient feature extraction method, BC was used to optimize the sensor with greater contributions and eliminate the tedious information of high-dimensional data from E-nose. According to our previous achievements, three kinds of artificial neural networks (ANN), including Naïve-Bayes network (NBN), radial basis function network (RBF), and random forest (RF), were used for the discriminant models of E-nose original and optimized responses.

Simple correlation was applied to figure out the relationship among the experimental results of sensory and instrumental analyses, namely, organoleptic evaluation, GC, and E-nose. Afterwards, scatterplots and line charts were generated to see if there is any linear correlation. In the end, principal components analysis (PCA) was used to classify the samples based on those three groups of data.

The statistical analyses were all performed applying SPSS version 22.0 (SPSS/IBM, Beijing, China; Licensing Certificate No. 20150408-LSBJ) and Weka software (free access at website: https://www.cs.waikato.ac.nz/ml/weka/).

## 3. Results and Discussion

Odor is one of the key elements for identification of TCM. Since the pros and cons of sensory and instrumental approaches, organoleptic evaluation by 20 trained panelists was firstly carried out and then morphological measurement, GC, and E-nose were used to offer further background for further verification, which means digital and objective information of volatiles compounds and aromatic fingerprint.

### 3.1. Organoleptic Evaluation

The descriptive statistics including samples' number, mean value, and minimum/maximum were obtained via Friedman Test (Table 1). The test statistics were as follows: Chi-Square is 219.259, degree of freedom is 24, and Asymp. Sig. is 0.000, which is far smaller than 0.05. That reminds us that the organoleptic evaluation results of 10 panelists are different and can be quite useful for Sha-Ren's identification.

According to logistic regression analysis with ordinal variables, the probability of each kind of samples could be forecasted, which is how many scores they will earn through this human panel. For instance, the total number of Yang-Chun-Sha from Guang-Dong province is 80 (5 + 15 + 32 + 28) as shown in Table 2. Their predicted probabilities of being scored at 7, 8, 9, and 10 should be 0.06, 0.19, 0.40, and 0.35, which were calculated from 5/80, 15/80, 32/8, and 28/80, respectively. In other words, if samples scored at 8, 9, and 10 were logically assumed to have better quality, then more than 90% of Yang-Chun-Sha samples from Guang-Dong would be considered as superior products (0.19 + 0.40 + 0.35 = 0.94). In contrast, Yang-Chun-Sha samples from Yun-Nan and Guang-Xi should be of average quality, because their total ratios of scores 4, 5, and 6 are 0.84 and 0.5, respectively. And the samples of the lowest quality are Lv-Qiao-Sha collected from markets, whose probability of scores 0, 1, and 2 is 0.82.

To summarize the results of organoleptic evaluation by 10 panelists, all 25 batches of Sha-Ren samples were divided into three groups, namely, high class (Yang-Chun-Sha from Guang-Dong), moderate class (Yang-Chun-Sha samples from Yun-Nan and Guang-Xi), and low class (Lv-Qiao-Sha from marketplaces). It resembles the concept of TCM experts that Yang-Chun-Sha from Guang-Dong is the geoauthentic medicine of better quality and clinic effects.

### 3.2. Morphological Measurement

**Figure 1:**
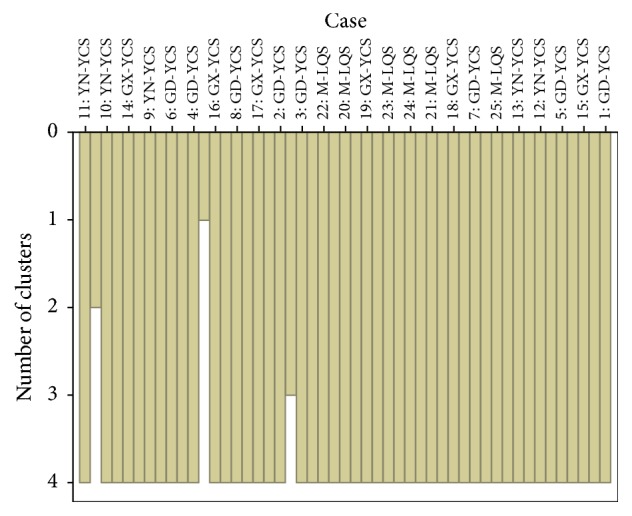
HCA vertical icicle diagram responses to samples based on morphological measurements with three indices (LD, TD, and 100 FW).

HCA applying furthest neighbor cluster method shows that all samples were classified into four groups (Figure 1). However, the results are confusable to a certain extent and not similar to organoleptic evaluation. It deserves to be noticed that all Lv-Qiao-Sha samples from markets were in the same group, though the classification of other samples was irregular.

To the next step, DA was used to find out whether these three morphological indices could help to get a satisfying identification of Sha-Ren samples. From the test of functions, the values of Wilks' Lambda are 0.295, 0.664, and 0.989, respectively. Sig. values of them are all higher than 0.01. Therefore, this discriminant analysis could not be able to separate the samples efficaciously, which stays on the same side of HCA results.

### 3.3. Determination of Bornyl Acetate via GC

Bornyl acetate in extracted samples was identified by comparison of their retention time with that obtained from the chromatograms of standard substance. The content of bornyl acetate in each sample was calculated using standard curve of bornyl acetate and illustrated in Table 3. According to the level of bornyl acetate contents, all samples could clearly be divided into three groups. The first one contains eight batches of numbers 1, 2, 3, 5, 7, 12, 13, and 15, whose values are all exceeding 3. The second one would be composed of batch numbers 4, 6, 8, 10, and 14, whose values are between 1 and 2. And the third one consists of 12 batches, namely, numbers 9, 11, and 16–25. From these results we could tell that most of Yang-Chun-Sha samples are of higher amount of bornyl acetate and all Lv-Qiao-Sha samples gathered themselves together, with lower bornyl acetate, which agrees with the results of morphological measurement. Hence herein it is proven that Lv-Qiao-Sha samples are inferior-quality products, which means their price would be too high.

### 3.4. BC Feature Extraction and Discriminant Models of Aromatic Fingerprint from E-Nose

In the estimation of discriminant models using three different classifiers (NBN, RBF, and RF), two validation methods were applied and distinguishing positive rate was calculated, which were tenfold cross-validation in the training set and external test set validation in the test set.

Through BA feature extraction method performed in Weka software, six MOG sensors, namely, S2, S3, S6, S8, S10, and S12, were selected to generate the optimum data set. However, based on this discriminant model, only Yang-Chun-Sha from Guang-Dong could be distinguished from the other samples successfully, which leads to two categories based on E-nose responses. It is in similar situation of organoleptic evaluation results, where it sheds light on the concept again that Yang-Chun-Sha is the geoauthentic medicine.

As shown in Table 4, distinguishing positive rates of three classifiers were all increased after BC feature extraction. In a word, this optimum data set of six sensors are containing enough effective information extracted from aromatic fingerprint from E-nose, which enables it to afford the identification task by eliminating the redundant data.

### 3.5. Correlation among Sensory and Instrumental Analysis of Sha-Ren's Odor

For the simple correlation analysis, the organoleptic analysis showed positive correlation with GC and E-nose, with correlation index of 0.650 and 0.807, respectively. However, according to the scatterplots and line graphs, no obvious linear regression was found. But it is easy to see that the curves of organoleptic analysis and GC are in the same trend.

**Figure 2:**
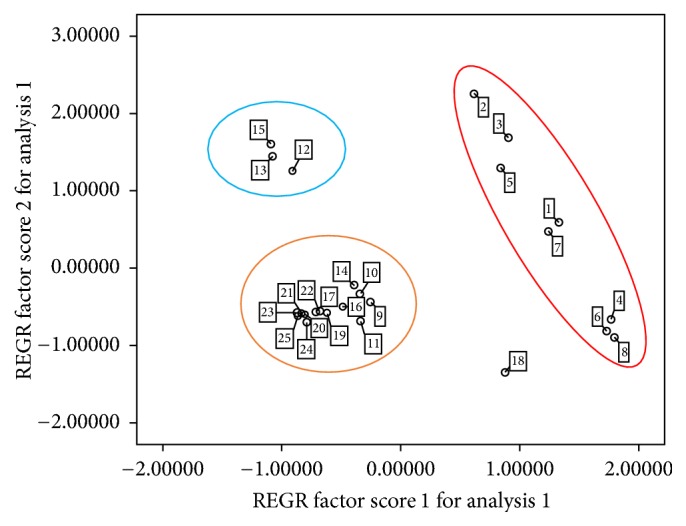
PCA score plot responses to Sha-Ren samples with PC1 and PC2.

As for PCA, samples were placed in three zones (Figure 2). These results are in great consistency with the results with organoleptic evaluation, though with subtle discrepancy. Furthermore, this model obviously provides much more details than E-nose applied alone, which could only differentiate the geoauthentic medicine from the others. That comes into a hypothesis: even E-nose is much more sensitive, but still there is some deeper information extracted by human panel, which have been searched out.

## 4. Conclusions

Twenty-five batches of Sha-Ren samples were collected from Guang-Dong, Guang-Xi, and Yun-Nan and marketplaces, which are Yang-Chun-Sha (*Amomum villosum* Lour.) and Lv-Qiao-Sha (*Amomum villosum* Lour. var.* xanthioides* T. L. Wu et Senjen) from* Zingiberaceae* family. For rapid and reliable authentication of confusable and of great price variance samples, sensory analysis was introduced to development a practical method. Based on a human panel with 10 trained assessors, organoleptic evaluation divided experimental samples into three classes: high class (Yang-Chun-Sha from Guang-Dong), moderate class (Yang-Chun-Sha samples from Yun-Nan and Guang-Xi), and low class (Lv-Qiao-Sha from marketplaces). For providing further material background and objective verification, three instrumental approaches were employed: morphological measurement with three indices (longitudinal diameter, transverse diameter, and 100-fruit weight), GC for determination of bornyl acetate contents, and E-nose for aromatic fingerprint. It is demonstrated in the results that GC and E-nose analyses were in great agreement with organoleptic evaluation, whilst morphological measurement nevertheless showed irregular classification. It could be explained that those three indices were not perfect and at least they did not represent the whole effective information. Further researches are in progress in our lab to look for better indicators and also improve the distinguishing positive rate of E-nose discriminant model.

## Figures and Tables

**Table 1:** Descriptive statistics via Friedman test of 25 samples by 10 panelists.

	*N*	Mean	Std. deviation	Minimum	Maximum
Sam. 1	10	9.50	.527	9	10
Sam. 2	10	8.90	.994	7	10
Sam. 3	10	9.40	.699	8	10
Sam. 4	10	9.50	.527	9	10
Sam. 5	10	8.10	.738	7	9
Sam. 6	10	9.00	.943	7	10
Sam. 7	10	8.80	.919	8	10
Sam. 8	10	9.10	.994	7	10
Sam. 9	10	5.40	.843	4	7
Sam. 10	10	5.10	.738	4	6
Sam. 11	10	4.30	.675	3	5
Sam. 12	10	5.10	.876	4	6
Sam. 13	10	4.50	.972	3	6
Sam. 14	10	5.00	1.054	3	6
Sam. 15	10	4.80	.919	3	6
Sam. 16	10	3.80	.919	2	5
Sam. 17	10	2.30	1.252	0	4
Sam. 18	10	2.50	.850	1	4
Sam. 19	10	2.80	1.135	0	4
Sam. 20	10	1.60	.843	0	3
Sam. 21	10	1.50	.707	1	3
Sam. 22	10	2.50	.707	1	3
Sam. 23	10	1.30	.949	0	3
Sam. 24	10	1.50	.972	0	3
Sam. 25	10	1.20	.919	0	3

**Table 2:** Cell information of logistic regression analysis with ordinal variables from 25 samples by 10 panelists.

Frequency		Score										
Habitats	Species											
		0	1	2	3	4	5	6	7	8	9	10
GuangDong	YangChunSha											
	Observed	0	0	0	0	0	0	0	5	15	32	28
	Expected	.000	.000	.000	.000	.000	.000	.000	5.000	15.000	32.000	28.000
	Pearson Residual	.000	.000	.000	.000	.000	.000	.000	.000	.000	.000	.000

YunNan	YangChunSha											
	Observed	0	0	0	2	16	19	12	1	0	0	0
	Expected	.055	.247	1.149	4.479	11.597	17.566	14.056	.850	.000	.000	.000
	Pearson Residual	−.235	−.499	−1.085	−1.228	1.475	.425	−.647	.164	.000	.000	.000

GuangXi	YangChunSha											
	Observed	2	2	10	16	14	10	6	0	0	0	0
	Expected	.471	2.036	8.077	18.863	18.222	8.972	3.215	.145	.000	.000	.000
	Pearson Residual	2.235	−.025	.727	−.796	−1.185	.372	1.597	−.381	.000	.000	.000

Market	LvQiaoSha											
	Observed	7	21	21	11	0	0	0	0	0	0	0
	Expected	8.280	19.830	20.631	8.329	2.171	.583	.168	.007	.000	.000	.000
	Pearson Residual	−.479	.321	.100	.997	−1.501	−.767	−.411	−.085	.000	.000	.000

Link function: Logit.

**Table 3:** Experimental results of Sha-Ren samples.

Batch number	Sample name	Species	Habitats	Score of human panel	Bornyl acetate content	S6-sensor response
(1)	GD-YCS	Yang-Chun-Sha	Guang-Dong	9.5	3.82	0.04
(2)	GD-YCS	Yang-Chun-Sha	Guang-Dong	8.9	6.64	0.04
(3)	GD-YCS	Yang-Chun-Sha	Guang-Dong	9.4	5.68	0.04
(4)	GD-YCS	Yang-Chun-Sha	Guang-Dong	9.5	1.74	0.04
(5)	GD-YCS	Yang-Chun-Sha	Guang-Dong	8.1	5.03	0.04
(6)	GD-YCS	Yang-Chun-Sha	Guang-Dong	9.0	1.52	0.04
(7)	GD-YCS	Yang-Chun-Sha	Guang-Dong	8.8	3.66	0.04
(8)	GD-YCS	Yang-Chun-Sha	Guang-Dong	9.1	1.35	0.04
(9)	YN-YCS	Yang-Chun-Sha	Yun-Nan	5.4	0.88	0.03
(10)	YN-YCS	Yang-Chun-Sha	Yun-Nan	5.1	1.06	0.03
(11)	YN-YCS	Yang-Chun-Sha	Yun-Nan	4.3	0.48	0.03
(12)	YN-YCS	Yang-Chun-Sha	Yun-Nan	5.1	3.71	0.03
(13)	YN-YCS	Yang-Chun-Sha	Yun-Nan	4.5	4.04	0.03
(14)	GX-YCS	Yang-Chun-Sha	Guang-Xi	5.0	1.24	0.03
(15)	GX-YCS	Yang-Chun-Sha	Guang-Xi	4.8	4.31	0.03
(16)	GX-YCS	Yang-Chun-Sha	Guang-Xi	3.8	0.80	0.03
(17)	GX-YCS	Yang-Chun-Sha	Guang-Xi	2.3	0.73	0.03
(18)	GX-YCS	Yang-Chun-Sha	Guang-Xi	2.5	0.71	0.04
(19)	GX-YCS	Yang-Chun-Sha	Guang-Xi	2.8	0.69	0.03
(20)	M-LQS	Lv-Qiao-Sha	Marketplaces	1.6	0.68	0.03
(21)	M-LQS	Lv-Qiao-Sha	Marketplaces	1.5	0.70	0.03
(22)	M-LQS	Lv-Qiao-Sha	Marketplaces	2.5	0.74	0.03
(23)	M-LQS	Lv-Qiao-Sha	Marketplaces	1.3	0.72	0.03
(24)	M-LQS	Lv-Qiao-Sha	Marketplaces	1.5	0.52	0.03
(25)	M-LQS	Lv-Qiao-Sha	Marketplaces	1.2	0.64	0.03

**Table 4:** Distinguishing positive rates of three classifiers (NBN, RBF, and RF) of original and optimum data set.

Cliassifer	Original data set		Optimum data set	
	Tenfold cross-validation	External test set validation	Tenfold cross validation	External test set validation
NBN	54	56	78	84
RBF	66	72	78	84
RF	64	76	78	84
